# Emergency Department Disposition Among Pediatric Road Traffic Injury Patients Admitted to AaBET Hospital Emergency Department, Addis Ababa, Ethiopia

**DOI:** 10.1155/emmi/8575907

**Published:** 2026-06-16

**Authors:** Ayalew Zewdie, Biruktawit Zemede, Nebiyat Semeredin, Zelalem Negash

**Affiliations:** ^1^ Emergency Medicine and Critical Care Department, St Paul’s Hospital Millenium Medical College, Addis Ababa, Ethiopia; ^2^ Emergency Medicine and Critical Care Department, African Health Science University, Kigali, Rwanda; ^3^ Internal Medicine Department, African Health Science University, Kigali, Rwanda; ^4^ Addis Ababa Medical and Business College, Addis Ababa, Ethiopia

**Keywords:** ED disposition, emergency department, outcome, pediatrics, road traffic injury

## Abstract

**Background:**

Children are particularly vulnerable to road traffic injuries (RTIs) leading to morbidity and mortality. Despite the high incidence of pediatric RTIs in Ethiopia, data on the patterns and consequences of pediatric RTIs are limited.

**Objective:**

To assess the characteristics and ED disposition among pediatric patients (age ≤ 18 years old) with RTI admitted to emergency department of Addis Ababa Burn, Emergency, and Trauma (AaBET) Hospital, Addis Ababa, Ethiopia.

**Methods:**

A hospital‐based cross‐sectional study was conducted, focusing on pediatric patients with RTIs who presented it to the emergency department of AaBET Hospital from December 12, 2021, to December 30, 2023, retrospectively. Data were collected using a standardized structured data collection checklist from hospital data records and medical charts. A sample size of 279 pediatric RTI patients was included from registered hospital records. After data cleaning, data were analyzed using SPSS Version 21. Descriptive statistics were used to describe the dependent variables.

**Result:**

Out of 279 patients, the median age was thirteen (IQR: 9–17). A total of 54.1% of the patients were male. One hundred and fourteen (40.9%) of those were from age 15–18. Two hundred and eight (74.6%) patients were pedestrians. Forty‐eight percent of the patients sustained injury while in transit to work or school. Seventy six (27.2%) came to the hospital with private cars or trucks, while 63 (22.6%) came with an ambulance. One‐hundred thirty five (48.4%) of the patients sustained extremity and pelvis injuries, while 41.9% sustained head injuries. Two‐hundred fifty‐five (91.4%) patients were discharged home from the emergency department. The emergency department mortality was 0.7%.

**Conclusion:**

In this study, children from the ages of 15–18 were more affected by RTIs. Pedestrians and while in transit to school or work were more injured. Head and extremity injuries were the predominant injuries that happened to children. Children and schools should be prioritized in targeted road safety initiatives, supported by stricter enforcement of traffic laws near school zones.

## 1. Introduction

Every year, around 1.19 million individuals lose their lives due to road traffic injuries (RTIs), which is the primary cause of death for individuals aged 5–29 years [1]. Surprisingly, 92% of these fatalities occur in low‐ and middle‐income countries, despite these countries having only 60% of the world’s vehicles [1]. Vulnerable road users, such as pedestrians, cyclists, and motorcyclists, account for over half of all road traffic deaths [1, 2].

Ethiopia, a country with a low vehicle/population ratio, is considered one of the country’s worst affected by RTIs [3]. This is due mainly to poor road safety plans, the failure of drivers to abide by the traffic rules, as lack of traffic enforcement, poor road design, lack of uptake of proven road safety policies and others [4].

According to a group of international and Ethiopian researchers and stakeholders, it is estimated that the annual mortality rate among pediatric under 5 years due to injuries will reach 11,989 by the year 2030 [5]. This represents a 12‐percentage point increase over a span of 15 years. Additionally, the number of deaths among pediatrics aged 0–14 years is projected to reach 30,364 by 2030(5).

Pediatrics are particularly vulnerable to RTIs, and these are influenced by multiple factors [6]. Pediatrics constitute a huge part of accidents, as studies have shown that they have lower danger detection ability and are more prone to impulsive actions in traffic. They struggle to assess the speed of approaching vehicles, often overestimating the distance [7]. In terms of cognitive development, pediatric pediatrics lack the ability to make safe decisions on the road, leading to unintentional risk‐taking, while older pediatrics and adolescents may actively seek out risky behaviors [8, 9]. Additionally, pediatrics’ smaller body mass makes them less visible to drivers, increasing the risk of multiorgan injuries due to their greater exposure [10].

The effect of RTI in pediatrics is beyond the immediate physical trauma. They will have different complications starting from early mortality to late disability.

Early Disposition from the Emergency Department is one of the indicators with the largest ED length of stay of 24 h. Those discharged from ED to home have lower injury severity and higher recovery. The likelihood of receiving medical care, the severity of injuries, and the frequency of hospitalizations are all higher in low‐income countries for pediatric traffic injury cases [11].

Despite the high incidence of RTIs in the nation, data on the patterns and consequences of pediatric RTIs are limited. Addis Ababa Burn, Emergency, and Trauma (AaBET) Hospital is a premier trauma center, but as of right now, it does not have thorough research that looks at the traits and results of pediatric patients with RTIs. The aim of this study was to assess the characteristics and ED disposition among pediatric RTI patients admitted to the emergency department of AaBET Hospital, Addis Ababa, Ethiopia. This study will provide baseline results that will help policymakers identify possible areas of improvement in pediatric trauma care.

## 2. Methods and Materials

### 2.1. Study Area and Study Period

Ethiopia is one of the most populated countries in sub‐Saharan Africa, around 110 million inhabitants. The country comprises 11 regions and two city administrations; 16% of its population lives in urban areas. AaBET Hospital is a part of St. Paul millennium medical college; it is the first and the only trauma center, founded in Ethiopia and established in 2014. It has emergency and critical care medicine and departments of orthopedic and traumatology, neurosurgery, general surgery, and plastic surgery. At full capacity, it serves as 250 beds patient load equivalent. The emergency department has Red zone with 5 beds, Orange zone with 12 beds, and Yellow Green zone with 40 beds. The ICU has a total of 11 beds. The emergency and critical care specialists run both the Emergency and ICU. It provides 24‐h 7‐day‐a‐week service with comprehensive laboratory and radiography services.

Patients used different modes to go to the Emergency Department like ambulance, taxi, public transport, or walking.

The study period was from December 2021 to December 2023, and the data collection period was from April to August 2024.

### 2.2. Study Design

A hospital‐based cross‐sectional study design was used using hospital registry and medical records from patients presenting to ED from December 2021 to December 2023.

### 2.3. Study Population

All pediatric (≤ 18 years old) RTIs admitted to AaBET hospital emergency department during the study period.

### 2.4. Inclusion and Exclusion Criteria

#### 2.4.1. Inclusion Criteria

Pediatric (≤ 18 years) RTI patients presented to emergency department from December 1, 2021, to December 30, 2023.

#### 2.4.2. Exclusion Criteria

Unknown disposition, incomplete documentation (> 30%), and repeat visit.

### 2.5. Sample Size

With the assumption of 95% confidence interval, a study in AaBET Hospital trauma pediatric patients showed that among pediatric RTI patients, 20.9% [12] of the patients had not discharged home, and with 5% margin of error, the sample size calculated using single proportion formula (*N* = *Z* ^2^p(1‐p)/d2), and adding 10% nonresponse rate, the total sample size of 279 was used.

### 2.6. Sampling Procedure

A total of 279 pediatric RTI patients who visited AaBET Hospital emergency departments between December 2021 and December 2023 were included from the ED registration book.

Their medical record number was used to extract the medical charts of the selected participants from the card room.

### 2.7. Data Collection Tools and Procedures

#### 2.7.1. Data Collection Procedures

Data were collected using standardized structured questionnaires from hospital registry and patients’ chart. The questionnaire was prepared based on previous studies [13, 14]. The checklist contains the following main categories of variables. Sociodemographic characteristics, type of injury and road user’s role, prehospital/scene care, ED diagnosis, ED treatment, and outcome/disposition. Two nurses collected the data after they were given training. The investigator supervised and trained data collectors. Data were collected using the questionnaire and filled in to Excel. The data collection tool was pretested before actual data collection.

#### 2.7.2. Operational Definition

➢ Pediatric RTI: RTI occurred in those ≤ 18 years old [12].
➢ ED disposition: emergency department final disposition.
   Discharged home: patients discharged home from the emergency department after evaluation and management.
   Not discharged: patients who died, admitted to ward, referred, or went against medical advice [14].

### 2.8. Data Processing and Analysis

The data were checked for completeness. The data collected were cleaned, coded, and entered to SPSS Version 21. Descriptive statistics were done and presented as tables and figures. The mean and standard deviation were calculated for the normality distributed data while median and interquartile range were calculated for skewed data. A logistic regression model was performed to generate hypothesis on factors that influence ED disposition.

## 3. Result

### 3.1. Sociodemographics

Two‐hundred seventy‐nine patients were analyzed in this study. The median age was 13 (IQR: 9–17). One hundred and fourteen (40.9%) were 15–18 years old. One‐hundred fifty‐one (54.1%) patients were male while 128(45.9%) were female. One‐hundred forty‐eight (53%) patients were from Addis Ababa, while 121(43.4%) were from Oromia. In relation to road users, 208 (74.6%) were pedestrians. One‐hundred thirty‐four (48%) patients sustained injury while in transit to work or school (Table 1).

**TABLE 1:** Characteristics of pediatric RTI patients at AaBET hospital emergency department, Addis Ababa, Ethiopia, from December 1, 2021, to December 30, 2023.

Variables	Category	Frequency	Percentage
Age median of thirteen (IQR: 9–17)	0–4	31	11.1
	5–10	69	24.7
	11–14	65	23.3
	15–18	114	40.9

Sex	Female	128	45.9
	Male	151	54.1

Address	Addis Ababa	148	53.0
	Oromia	121	43.4
	Amhara	5	1.8
	South	3	1.1
	Afar	2	0.7

Road user	Pedestrian	208	74.6
	Passenger	42	15.1
	Driver	20	7.2
	Unknown	9	3.2

Situation	While in transit to work or school	134	48.0
	While engaged in leisure activity	30	10.8
	While working for income	26	9.3
	Others (cross road, travel, sport activity)	13	4.7
	Missing	76	27.2

### 3.2. Clinical Characteristics

The majority (60.6%) of the patients did not receive care at the scene, but this variable was missing for a large number (103, 36.9%). One‐hundred twenty‐two (43.7%) patients were referred to AaBET Hospital while 157 (56.3%) visited the emergency department directly without referral. Of those referred patients, 84 (68.9%) came from primary health centers and 28(23.0) were from first‐level hospitals. Seventy six (27.2%) came to hospital in private car or truck while 63 (22.6%) came with ambulance. From those referred, 49/122 (40.2%) used ambulance; while from those who came without referral, 14/157(8.9%) came with ambulance. Two‐hundred seventy‐four (98.2%) cases GCS were from 14 to 15. One‐hundred thirty‐five (48.4%) patients sustained extremity and pelvis injury while 117(41.9%) sustained head injury. One‐hundred sixty six (59.5%) had laceration or wound followed by contusion, hematoma, or abrasion, which accounts for 66 (23.7%) (Table 2).

**TABLE 2:** Clinical characteristics and prehospital status of pediatric RTI patients at AaBET hospital emergency department, Ethiopia, from December 1, 2021, to December 30, 2023.

Variables	Category	Frequency	Percentage
Prehospital care	No scene care delivered	169	60.6
	Care by healthcare provider	6	2.2
	Care by layperson	1	0.4
	Missing	103	36.9

Referral status: referred	Referred	122	43.7
	Self	157	56.3

Level of referring facility	Primary health center	84/122	68.8
	First‐level hospital	28/122	23.0
	Second‐level hospital	8/122	6.6
	Tertiary hospital and higher level	2/122	1.6

Mode of arrival	Private car, truck	76	27.2
	Taxi	70	25.1
	Ambulance	63	22.6
	Walking	45	16.1
	Others	25	9.0

GCS	≤ 8	4	1.4
	9–13	1	0.4
	14–15	274	98.2

Body site injury	Head and neck, spinal	117	41.9
	Chest	11	3.9
	Abdomen	6	2.2
	Extremity, pelvis	135	48.4
	Soft tissue	7	2.5
	Missing	3	1.1

Type of injury	Laceration/wound (not amputation)	166	59.5
	Contusion, hematoma, or abrasion	66	23.7
	Fracture	30	10.8
	Blunt trauma	6	2.2
	Joint dislocation, sprain	5	1.8
	Pneumothorax or hemothorax	1	0.4
	None	2	0.7
	Missing	3	1.1

### 3.3. Treatment

Two‐hundred sixty‐eight (96.1%) patients received analgesics, 169 (60.6%) got tetanus vaccine, 126 (45.2%) got antibiotics, and 108 (38.7%) received IV fluids (Table 3).

**TABLE 3:** Treatment and ED disposition of pediatric RTI patients at AaBET Hospital emergency department, Addis Ababa, Ethiopia, from December 1, 2021, to December 30, 2023.

Procedures and treatment	Analgesia	268	96.1
	Tetanus	169	60.6
	Antibiotics	126	45.2
	IV fluids	108	38.7
	Glucose	60	21.5
	Wound closure laceration repair	8	2.9
	Noninvasive extremity traction splint	8	2.9
	Fracture reduction splinting	5	1.8
	Oxygen	3	1.1
	Endotracheal intubation ETT	2	0.7
	Mechanical ventilation	2	0.7
	Urinary catheterization	1	0.4
	Blood products	1	0.4

Disposition	Discharged home	255	91.4
	Admitted operating theater	11	3.9
	Transferred to another hospital	5	1.8
	Admitted ward	2	0.7
	Admitted ICU	2	0.7
	Died	2	0.7
	Went Against Medical Advice (WAMA)%	1	0.4
	Missing from chart	1	0.4

Two‐hundred fifty‐five (91.4%) patients were discharged home from the emergency department. Eleven (3.9%) patients were admitted to operation theater (Table 3).

Overall, 91.4% of the patients were discharged home from emergencies, while only 8.6% were not discharged (Table 4).

**TABLE 4:** ED disposition among pediatric RTI patients at AaBET Hospital emergency department, Addis Ababa, Ethiopia, from December 1, 2021, to December 30, 2023.

Variables	Category	ED disposition		COR(CI)	*p* value
		Not‐discharged home *n* (%)	Discharged home *n* (%)		
Sex	Female	8 (33.3%)	120 (47.1%)	1	
	Male	16 (66.7%)	135 (52.9%)	1.77 (0.73–4.30)	0.20

Address	AA	6 (25.0%)	142 (55.7%)	1	0.007^∗^
	Out of AA	18 (75.0%)	113 (44.3%)	3.77 (1.449–9.811)	

Road user	Pedestrian	15 (62.5%)	193 (75.7%)	1.868 (0.779–4.478)	0.161
	others	9 (37.5%)	62 (24.3%)	1	

Mode of arrival	Ambulance	13 (54.2%	50 (19.6%)	4.845 (2.050–11.455)	0.000
	Others	11 (45.8%)	205 (80.4%)	1	

Body site injury	Head injury	14 (58.3%)	103 (40.4%)	2.066 (0.884–4.830)	0.094
	Another site	10 (41.7%)	152 (59.6%)	1	

^∗^ *p* < 0.05.

## 4. Discussion

This is the first research conducted at the AaBET Hospital to evaluate the characteristics and emergency department disposition for pediatric patients who have had RTIs.

Approximately 41% of the 279 individuals that were investigated were between the ages of 15 and 18. This study is comparable to a study from Qatar (56.4%) [15]. Pediatrics at this age are actively transitioning into adolescents; as such, they may be more exposed to dangerous driving situations, take part in riskier activities, or have less supervision, all of which enhance their susceptibility.

In this study, male were more affected than female, which is around 54%, but this is lower than other studies, which is more than 70% [16–19].

The largest impacted group was pedestrians, at 75%, which is comparable to earlier research but an even greater proportion [18, 20]. Pedestrians are more likely to be involved in accidents, which may be caused by unsafe driving or pedestrian conduct, a lack of protective gear, or excessive traffic volumes. In contrast, just 15% of the participants in the UAE research were pedestrians. This discrepancy may result from a variety of contextual and geographical variables, including the built environment, traffic patterns, transportation infrastructure, and even variations in driving habits and safety laws. Pedestrian safety protocols around schools are needed to decrease pedestrian injuries. A greater percentage of children sustained injuries while traveling to or from school or work. This demonstrated that the issue may be with drivers or pedestrians disobeying traffic laws, with speed limits near schools, or with heavy traffic in the vicinity of workplaces.

A higher percentage of children did not receive any scene treatment. This is similar to the study from Jimma medical center; more than half of RTI patients did not get any scene care [14]. This calls for community involvement in first aid training, raising awareness, improving access to first responders, and implementing prevention and first aid classes in schools.

Children who visited the hospital straight without a referral made up a larger percentage. Since it is the primary trauma facility in the community, families may bring their children straight to the emergency room, which may explain why a larger percentage of youngsters from Addis Ababa are here. Referral linkage should be used to manage resources and give the best treatment possible to the patients who are in need since this results in congestion in the ED. From the referred ones, most came from primary health center directly.

Around 50% of the patients came with private cars and taxis. Only 22% came with ambulances. From those referred, 49/122 (40.2%) used ambulance; while from those who came without referral, 14/157(8.9%) came with ambulance. There is very low use of ambulance for bringing patients from the scene, which is like previous studies in the country [21, 22]. Apart from this, even those referred from health facilities, only 40.2% came using an ambulance. This requires further study to assess why pediatric patients are not sent using ambulances from previous health facilities. Improving the emergency medical service is crucial to provide timely care at the scene and while transporting to health facility.

Extremity injuries followed by head injuries were the predominant injuries that pediatric patients sustained, which is like most studies [17, 18, 23]. Higher head injuries are particularly worrisome since, if not treated quickly and effectively, they may have serious and even fatal neurological effects. But most of pediatrics had mild traumatic injuries with GCS 14‐15. Substantial proportion of pediatrics sustained laceration or wounds followed by contusion or hematoma.

Stabilizing the patient using this structured method is critical for immediately managing any immediately life‐threatening problems [24, 25]. In this study, only 3 patients were on oxygen and only 2 patients were on definitive air way management and on mechanical ventilator (0.7%). Around 40% of the pediatric patients received fluid management.

Pain management is also one of the crucial cares for trauma patient [26–28]. Most patients received analgesics.

Very high proportion of pediatric patients was discharged home from the emergency department. This is similar to studies from Turkish and Guinea [18, 29]. This shows those discharged from ED to home most likely have low injury severity and higher recovery or because of lack of bed in wards patients stayed in emergencies and discharged home, which requires further study.

The ED morality was very low, which is around 0.7% lower than Turkey (4.4%), Qatar (3.4%), and Nigerian (9.4%) [15, 18, 23].

### 4.1. Limitation of the Study

The study has several limitations. The first one is the study is descriptive, which requires further study to assess determinant factors for ED disposition The second one is the results may not apply to the entire nation because this is single hospital cross‐sectional research multicenter study that is recommended for generalization. This study did not examine other variables, such as the extent of the injury, the timelines of treatment, or the availability of resources. Lastly, the missed data may create selection bias for generalization. We suggest that further study is needed with a larger sample size and multisite for generalization of the factors that influence the ED disposition.

## 5. Conclusion

In this study, children from age 15–18 were more affected by RTI. Pedestrians and while in transit to school or work were more injured. Head and extremity injuries were the predominant injuries that happened to pediatric patients. Most patients in this study were discharged home. Children and schools should be prioritized in targeted road safety initiatives, supported by stricter enforcement of traffic laws near school zones.

## Author Contributions

Ayalew Zewdie, Biruktawit Zemede, Nebiyat Semeredin, and Zelalem Negash have all contributed equally to the conception of the work, including the acquisition, analysis, or interpretation of data, and drafting and revising

## Funding

There was no funding for this study.

## Disclosure

All authors have approved the final version to be published and agreed to be accountable for all aspects of the work.

## Ethics Statement

The study was conducted in accordance with the Helsinki Declaration. Written legal ethical clearance was obtained from the Addis Ababa Medical and Business College (AAMBC) Institutional Review Board (IRB) AAMBC/STU/20251, and a support letter was provided to AaBET Hospital from AAMBC to facilitate data collection. Although minors were included in the study, the Addis Ababa Medical and Business College (AAMBC) Institutional Review Board (IRB) waived the requirement for informed consent from parents or legal guardians, as we conducted a record review using anonymous data.

## Consent

Please see the Ethic Statement.

## Conflicts of Interest

The authors declare no conflicts of interest.

## Supporting Information

Additional supporting information can be found online in the Supporting Information section.

## Supporting information


**Supporting Information** STROBE CHECKLIST: attached under supporting.

## Data Availability

The data used to support the findings of this study are available from the corresponding author upon reasonable request.
